# AI for social good and the corporate capture of global development

**DOI:** 10.1080/02681102.2023.2299351

**Published:** 2024-02-16

**Authors:** Gianluca Iazzolino, Nicole Stremlau

**Affiliations:** a Global Development Institute, The University of Manchester, Manchester, UK; b Centre for Socio-Legal Studies, Faculty of Law, University of Oxford, Oxford, UK; c School of Communication, University of Johannesburg, South Africa

**Keywords:** AI4SG, Big Tech, ICT4D, political economy

## Abstract

This article focuses on the AI for Social Good (AI4SG) movement, which aims to leverage Artificial Intelligence (AI) and Machine Learning (ML) to achieve the United Nations Sustainable Development Goals (UN SDGs). It argues that, through AI4SG, Big Tech is attempting to advance AI-driven technosolutionism within the development policy and scholarly space creating new opportunities for rent extraction. The article situates AI4SG, within the history of ICT4D. It also highlights the contiguity of AI4SG with the so-called 4th Industrial Revolution (4IR), a framework that places AI and other digital innovations at the center of national and international development and industrial policy agendas. By exploring how Big Tech has attempted to depoliticize datafication, we thus suggest that AI4SG and 4IR are mutually reinforcing discourses that serve the purpose of depoliticizing the development arena by bestowing legitimacy and authority to Big Tech to reshape policy spaces and epistemic infrastructures while inserting themselves, to an unprecedented degree, between the citizen (data) and the state (development and policy).

## 1. Introduction

Recent years have seen a booming number of initiatives leveraging digital technologies to address the United Nations Sustainable Development Goals (SDG) agenda (Cossy-Gantner et al., 2018; Gwagwa et al., 2020; Sætra, 2022; Stein, 2020; Vinuesa et al., 2020). These often-hyped interventions, endorsed by international and aid organizations (United Nations, 2018), reflect the widespread diffusion of digital tools and the growing availability of big data across the Global South (Heeks, 2016; Kshetri, 2014; Taylor & Broeders, 2015; Walsham, 2017). At the same time, this emphasis on data-driven technologies applied to the SDGs signals a shifting ‘technopolitics of development’ (Fejerskov, 2017), increasingly influenced by tech firms and, specifically, US-based Big Tech corporations such as Google, Meta, and Microsoft (henceforth only Big Tech).^1^ This shift is particularly evident in the so-called AI for Social Good (AI4SG) movement, which aims to leverage Artificial Intelligence (AI) and Machine Learning (ML) to achieve the SDGs (Cowls et al., 2021; Schoormann et al., 2021; Tomašev et al., 2020).

In this article, we argue that the corporate attempt to advance AI-driven technosolutionism through the AI4SG movement has implications for the way policymakers, scholars, practitioners and community-based organizations do and think of development. While being a catchy label, AI4SG remains a fuzzy concept (Shi et al., 2020). Through a ‘top-down approach that presupposes what good is’ (Madianou, 2021, p. 854), it attempts to deflect criticism by spilling over into a moral sphere and transcending the domain of development and, eventually, politics. AI4SG is grounded in a culture of ‘humanitarian neophilia’ (Scott-Smith, 2016), in which an ‘optimistic faith in the possibilities of technology’ is combined ‘with a commitment to the power of markets’ (4). Henriksen and Richey (2022) argue that ‘AI4SG (as a material and discursive phenomenon) frames controversial and profitable data practices as having public value and thereby obscures the power relations and politics of digital capitalism’ (26). In his political economy analysis of AI and environmental sustainability, Dauvergne (2020) concludes that ‘eco-business is not endeavouring to advance social justice or to protect the earth but is aiming to expand markets, sales and corporate power’ (15).

We specifically contribute to this line of criticism by situating AI4SG, on the one hand, within a longer history of corporate power, technology, and development that traces back to the information and communication technologies for development (ICT4D) movement; and, on the other, by highlighting the contiguity of AI4SG with the so-called 4th Industrial Revolution (4IR), a framework that places AI and other digital innovations at the center of national development and industrial policy agendas. Spearheaded by the World Economic Forum (WEF) (Butcher et al., 2021; de Ruyter et al., 2018), the notion of 4IR refers to the ‘integration between the digital, biological, and physical worlds’ (Butcher et al., 2021, p. 16) facilitated by the widespread use of emerging technologies (including AI) for advancing socio-economic transformations. This aggregation of digital technologies is expected, in very broad strokes, to refashion modes of production and social reproduction, including the articulation of capital and labor, and the relationship between national states and the private sector (Morgan, 2019). Embraced by international organizations such as the World Bank (WB) and the Organization for Economic Cooperation and Development (OECD), 4IR overlaps with, and in some countries, such as South Africa, is being used in public debate as a placeholder for AI4SG. Our argument is that AI4SG and 4IR are mutually reinforcing discourses that serve the purpose of depoliticizing the development arena by bestowing legitimacy and authority to Big Tech to reshape policy spaces and epistemic infrastructures.

While we agree with Bjola (2021), who argues that AI could change how ‘development challenges are identified, studied, and managed’ (2), it is paramount to unpack the power relationships behind the AI assemblage. In our analysis, we draw on Tania Murray Li’s (2007) articulation of problematisation and ‘rendering technical/rendering nonpolitical’ as key practices to ‘translate the will to improve into explicit programs’ (7) to suggest that the conversation around AI4SG is premised upon a process of depoliticization through datafication. In doing so, we show how deploying AI and big data to address the SDGs is another ‘weapon in the armoury of the ‘anti-politics machine’ constituted by the discourses and practices of development’ (Harriss, 2002, p. 112). The twin discourses of AI4SG and 4IR give prominence to corporate actors and elevates technical expertise to frame a developmental issue and address its solution. However, depoliticization as the process of removing ‘the political character of decision-making’ (Burnham, 2001, p. 128) is, in itself, a political act. In our article, we point to the current trend to leverage corporate-driven AI and datafication to render opaque both the production of evidence on which policy-making is based, and the processes to implement these policies. This strategy is pursued, we suggest, by influencing the policy space and reshaping epistemic infrastructures and practice.

We are cautious about sweeping generalizations, particularly as Western (mainly US-based) and Chinese digital tech firms deploy very different strategies as they vie for influence and market shares in the Global South, especially Africa, the primary geographic focus of our study. Comparing their approaches is beyond the scope of this article, and we are mainly concerned with the former to suggest that the libertarian ideology (Dignam, 2020) of Western tech giants is percolating into development strategies and discourse, with mixed results.

This article draws on more than 30 interviews with donors, policymakers, humanitarian practitioners, private sector representatives and tech firm executives conducted between 2017 and 2022^2^ face-to-face in Kenya, Rwanda, and South Africa, and remotely with key informants in the US, Switzerland, Germany, and Belgium. The three African countries are countries where many AI4SG initiatives across multiple sectors have been rolled out over the past ten years. Moreover, the backdrop of this study is inspired by insights derived from dozens of interviews conducted by the authors for different research projects on digital technologies and governance over more than two decades.

We begin by locating our research within the historical trajectory of technology companies attempting to drive the development agenda in the Global South. For more than half a century there has been a line of research within development studies and communications studies on the potentially modernizing effects of media (whether radio, print or television) that later accelerated with discussion around ICTs for Development with an emphasis on how everything from the internet to mobile phones can help countries ‘leapfrog’ development. Our article reflects on the continuity of this debate with the current turn to AI. We explore this turn, both in discourse and practice, through three main streams of analysis – policy, infrastructure, and datafication.

Policy refers to efforts by corporate actors to influence and shape national and international policy in such a way that is favorable to their business interests and political (and ideological) priorities. Infrastructure explores how these same corporate actors have long been actively building proprietary infrastructures to both extend access to their platforms, and attract more users, while also harvesting more data. This leads to the third area of datafication, where companies are increasingly moving into the business of prediction- harvesting, justifying, and using large amounts of data to attempt to address and aid international organizations as part of an effort to address, or pre-empt, crises in advance, a practice referred to as ‘anticipatory action.’ We conclude by arguing that the acceleration of corporate influence in the AI4D agenda in these three areas- policy, infrastructure, and datafication- not only reshapes how government, international organizations and policy actors conceptualize development, in the process skewing indicators and values, but it also fundamentally shifts the discourse and language to one that favors the vision and priority of these corporate actors rather than that of the public sector, the nation-building projects of the state, and the values and priorities of citizens.

## 2. Technologies, corporate power and the history of development in Africa

In her essay on governmentality and development in Indonesia, Murray Li (2007) highlights the connection between the framing of development challenges and the range of solutions that can be identified to address them. These are steps that can lead to the practice of ‘rendering technical,’ which defines the experts and ‘constitutes the boundary between those who are positioned […] to diagnose deficiencies in others, and those who are subject to expert direction’ (11). It is, she argues, ‘a boundary that has to be maintained and that can be challenged’ (12). The implication of ‘rendering technical,’ in Murray Li’s analysis, is the obfuscation of politics, or ‘rendering nonpolitical,’ as ‘(f)or the most part, experts tasked with improvement exclude the structure of political-economic relations from their diagnoses and prescriptions’ (Murray Li, 2007).

The current involvement of the private sector in the technopolitics of development, and specifically Big Tech, differs substantially from the role that it took during the 1980s (Mann & Iazzolino, 2021). Back then, the privatization of public assets was part of a larger package of policies to curb local government regulation and social spending, remove government subsidies and price control, liberalize trade and devaluate local currencies (Mkandawire & Soludo, 1999). Today, the state is the primary interlocutor and client of tech companies building the material infrastructure through which data are constituted as a resource for governance and development. Within this emerging digital development paradigm, tech firms are accruing power in the form of state overreliance on their services, or what Busemeyer and Thelen (2020) call ‘institutional source of business power.’ Besides redefining state technopolitics through the design, management and maintenance of data infrastructures, tech firms are also building their own legitimacy by influencing the state’s regulatory approach to data collection and usage across multiple fields of application. Through industry associations like the Groupe Speciale Mobile Association (GSMA), international organizations such as United National Capital Development Fund (UNCDF), or think tanks like the Consultative Group to Assist the Poor (CGAP), tech firms and philanthro-capitalist actors are advancing narratives of digital development and advising policymakers and regulators on how to shape regulatory frameworks, officially for maximizing the inclusive potential of digital technologies but, in fact, for carving new spaces of value extraction for private companies.

To understand the influence of Big Tech on the current policy and practitioner discourse on digital technologies and development in Africa, we will briefly trace the transformation of the relationship between development and digital technologies over the past 40 years and the prominence that tech companies have acquired over this period. Information and communication technologies (ICTs) were first embraced by international organizations, NGOs institutional donors, and national governments in low- and middle-income countries to support development interventions on specific issues (Heeks, 2009). More recently, though, corporate actors have taken the lead in going beyond siloed solutions to developmental challenges, and focus instead on the expansion of data infrastructures in Africa (Mann, 2018; Taylor & Broeders, 2015). Their hegemony mainly rests on crafting a digital development discourse that has been largely legitimized by policymakers, donors, development practitioners, and, to some degree, academics.

Since the 1980s, tech firms have increasingly penetrated markets in the Global South on the heels of the popularity of the personal computer first, and later the internet and mobile phones. Even before that, though, corporations like IBM and Hewlett Packard have been instrumental in shaping technopolitics by enabling the state to expand its infrastructural power. For instance, a 1986 report by anti-apartheid activist Richard Knight to assess the potential impact of international boycotts on Pretoria’s minority rule shed light on how ‘American computer companies have in particular played a strategic role in providing equipment and technology that has directly bolstered the apartheid system’ (Knight, 1986, p. 2). Moreover, foreign computing firms supplied mainframes to state agencies and state-owned companies in countries like Nigeria for supporting the national census (Idowu et al., 2008) or Kenya for improving payroll systems (Francis, 2015). In this phase, public administrations were the clients of tech corporations. The technology provided by these latter did not have explicit developmental purpose, although it was deployed to improve the efficiency of the public administrations (Heeks, 2009) and, in general, to support the state in implementing its ideological goals, whether based on economic growth, as in case of newly-independent countries, or apartheid, as in the case of South Africa. As we will argue later, datafication substantially changed this relationship, blurring the boundary between ICTs in the Global South and ICT for 4D and strengthening the negotiating position of tech corporations. They have done so, we suggest here, by couching their initiatives in developmental terms. The legacy of ICT4D has thus provided Big Tech with an opportunity to capture donor- and state-driven development agendas initially through corporate social responsibility programs and then by promoting digital development as an overarching policy and practitioner discourse premised upon the belief in the power of digital technologies to increase the efficiency and efficacy of development interventions.

Throughout the 1990s, the convergence of ICTs and development was driven by the mission to overcome the ‘digital divide,’ or the access gap to the internet between the Global North and the Global South (Gagliardone, 2020), strengthen civil society organizations, and improve state accountability under the auspices of the ‘good governance agenda’ (21). As effectively summarized by Heeks (2009),

(t)he digital technologies of the 1990s, then, were new tools in search of a purpose. Development goals were new targets in search of a delivery mechanism. That these two should find each other and fall in love was not unexpected. (p. 4)

With the formalization of the Millennium Development Goals (MDGs) in 2000, international development organizations and NGOs became instrumental in shaping the trajectory of ICT4D, while the private sector remained on the margins, providing support to pioneering NGOs such as Computer Aid, established in 1996 to provide refurbished PCs from the UK to educational and civil society organizations in the Global South. Between the late 1990s and the early 2000s, the discussion on ICT4D was informed by publications like the World Bank’s 1998 World Development Report ‘Knowledge for Development’ and events such as the World Summits on the Information Society held in Geneva in 2003 and Tunis in 2005. African policymakers first showed interest in developing ‘an action plan on ICTs to accelerate socio-economic development in Africa’ (UNECA, 2008) by launching the African Information Society Initiative (AISI) in 1996 following a resolution of the UN Economic Commission for Africa (UNECA) Conference of Ministers. By the early 2000s, tech companies started being more conspicuous in the ICT4D space by supporting national development agencies and NGOs with initiatives like ‘One laptop per child,’ launched in 2005, or Telecentre.org, established in San Francisco, California, in 2005 and incubated by Microsoft, Canada’s International Development Research Centre, and the Swiss Agency for Development and Cooperation to expand access to ICTs and the internet across emerging economies. A multidisciplinary scholarship sought to capture the social implications of these ICT4D initiatives, focusing on how technology and knowledge were transferred and adapted to local contexts, the processes of social embeddedness of technology and the techno-organizational transformations reflecting global political and economic changes (Avgerou, 2008). It was Prahalad’s concept of ‘bottom of the pyramid’, first popularized in 2004 and referring to the three billion on the planet living on an average of less than US$ 2 per day, that gave a boost to the role of the private sector within development circles (Dolan, 2012). As development agencies and institutional donors, including DFID (2005), embraced an approach to poverty reduction based on the principle of ‘building a market that works for the poor,’ corporations started making inroads in these markets by partnering with informal entrepreneurs and NGOs (Webb et al., 2010).

The penetration of tech firms gained momentum after 2010 when a clear business case started emerging driven by the dramatic increase in mobile phone subscriptions, leaping from 87 million to 700 million in seven years, between 2005 and 2012 (ITU, 2013). It was however the boom of mobile money platforms, first in Kenya (Kusimba, 2021) and then, gradually, across the rest of the continent, that attracted the interest of a more variegated range of tech actors, keen to explore business approaches at the intersection of technology and finance. The post-2015 development agenda endorsed the private sector as a development agent and explicitly called for private investors to fund the SDGs (Mawdsley, 2018; Rashed & Shah, 2021). The same period also witnessed a major leap forward in AI research (Mann et al., 2020), thus placing Big Tech at the center of this revolution.

In the next section, we discuss how the ‘corporate socio-technical imaginary’ (Hockenhull & Cohn, 2021) around AI, and buttressed by substantial investments and lobbying efforts, has percolated into development discourses.

## 3. AI rises

As Katz (2020) points out, AI has been, since the birth of the concept in the 1950s, ‘animated by, and has in turn refuelled, the imperial and capitalist projects of its patrons’ (4). Initially devised in the context of the Cold War as a tool to gain a strategic advantage over the USSR and advance US imperial agenda, AI has remained imbued with an ‘ideology of whiteness,’ seeping into the field’s composition and epistemologies (Katz, 2020, p. 154). After a steep decline in research and investments in the second half of the 1980s and early 1990s (the so-called ‘AI winter’), interest picked up again at the turn of 2000s, and particularly from 2012, when the neural network, or ‘deep learning,’ architecture developed by Geoffrey Hinton won an international computer vision contest (Krizhevsky et al., 2012). Moving away from the hitherto dominant rule-based approach to symbolic knowledge to mimic the structure of the human brain, neural networks, also known as ‘narrow AI,’ relied on data sets to recognize patterns on the basis of variables, or parameters, and thus ‘make specific predictions […] based on quantifying probability’ (Pasquale, 2020, p. 55).

It is against this backdrop that initiatives such as Project Lucy were launched. Incubated in Nairobi’s IBM Research Lab and unveiled in March 2014, the project was meant to showcase the potential of AI for the ‘rich, varied language of health care, finance, law and academia’ (Lohr, 2021), by adapting a supercomputer developed by IBM Watson, the AI division of the US tech giant, and first presented in 2011 in the US TV quiz show Jeopardy!. ‘Project Lucy,’ thus named after the eponymous 3.2-million-year-old remaining of a female hominid found in the Rift Valley, was designed to ‘marry together cognitive computing and problems of Africa’ (Okune, 2020). However, since the onset Project Lucy was bogged down by over-ambitious goals and the challenge to align corporate social responsibility (CSR) concerns and the need to sustain the corporation’s portfolio in Kenya and South Africa.^3^ Also, its conjectural approach raised unanticipated practical and ethical issues. For instance, asked to find a solution to the patchy data on school attendance, IBM Lab researchers ‘played around with how we can build a face recognition technology that basically allows us to identify exactly who goes to school.’^4^ Even before the pilot was launched, though, this idea was marred by a broad range of challenges, such as the opposition of the school leadership to the installation of the scans because the funding each school received depended on the number of students, the technical unreliability of the scanning and the reluctance of the students, who liked this biometric roll call to surveillance.

Despite its early demise, Project Lucy was the harbinger of a trend that, in later years, would see the erosion of the boundary between CSR and business goals as tech corporations, either directly or indirectly (through foundations), leveraged data-driven projects to cultivate relationships with policymakers, display the potential of AI/ML to the burgeoning Africa business process outsourcing (BPO), agribusiness and financial sectors, and test predictive models. This trend intensified as a rhetoric of disruption spilled over from the tech world into the development sector. In fact, the notion of ‘disruptive technologies’ traces back to 1995, when it first appeared in an article by Bower and Christensen (1995) in the Harvard Business Review, and became the buzzword through which Silicon Valley tech giants presented their mission to the world as their public visibility and capitalization increased. The World Bank officially embraced the category of Disruptive Technologies for Development (DT4D), which includes AI, in 2018 when, in partnership with Credit Suisse, it launched the ‘Disruptive Technologies for Development Fund.’ In the press release of the launch, the World Bank Group President Jim Yong Kim thus commented on the initiative:

The urgency of the challenges around us – from climate change to forced displacement – requires a re-think of strategic partnerships […] Collaborating with new partners to end poverty will help us make innovative use of technology and maximize finance for development. (World Bank, 2018)

The DT4D fund spurred a competition to identify innovative applications of disruptive technologies and eventually a program. It also contributed to carving a space in which governments, NGOs, tech firms and development agencies discussed potential partnerships. Yet, as Bjola (2021) points out, when it comes to how AI would disrupt the field of development, ‘the object of digital disruption (what is being disrupted?), mode (how is being disrupted?), and effect (what are the consequences of disruption?) have largely remain unquestioned thus far’ (8). The expression of this multisector, interdisciplinary and explicitly SDGs-driven conversation is the AI4SG movement.

This field of practice is shaped by win-win narratives on the potential of synergies between business and development/humanitarian stakeholders to achieve the SDGs. A review of AI4SG projects by Cowls et al. (2021), though, shows their uneven distribution across the SDG agenda, with the overwhelming majority of projects addressing SDG 3 (‘Good Health and Well-Being’), followed by SDG 12 (‘Responsible consumption and production’) and SDG 13 (‘Climate action’), while SDGs 5 (‘Gender Equality’), 16 (‘Peace, Justice and Strong Institutions’), and 17 (‘Partnerships for the Goals’) draw significantly less attention. Moreover, this and other works (Schoormann et al., 2021; Vinuesa et al., 2020) point out that these initiatives have yielded mixed results, proving often ethically problematic because the threats generally associated with AI/ML might be exacerbated in economically and politically fragile contexts. The overreliance on AI systems could indeed contribute to the reproduction of structural inequalities and injustice built into the data sets used to train predictive and generative models (Bender et al., 2021; Birhane & Prabhu, 2021; Eubanks, 2018; Holzmeyer, 2021). This risk is compounded by the cost-cutting logic underpinning the data collection and annotation processes that are central to machine learning (Jo & Gebru, 2020) and by the opacity of the system (Burrell & Fourcade, 2021), which hinders accountability.

Moreover, the popularity of AI has coincided with the increasing influence of the tech industry over AI research and ethics agenda (Gerdes, 2022) through massive hiring of AI scientists, computing power, and large datasets (Ahmed et al., 2023). Big Tech in particular has positioned itself as the main force shaping the research trajectory and the policy and popular conversation around AI by leveraging not only technical and financial resources, but also its geopolitical clout (Kak & Myers West, 2023). While the academic attention on the political economy and policy relevance of Big Tech is growing (Birch & Bronson, 2022; Khan, 2016; Moore & Tambini, 2021), the implications of Big Tech’s attributes – network effects, winner-takes-all dynamics, and financial leverage (Birch & Bronson, 2022, p. 3)– on the global development arena has hitherto been largely overlooked. However, as we suggest in this article, the two key dimensions of Big Tech – scale/scalability and platformization – (Birch & Bronson, 2022), which set current US-based tech giants apart from past tech firms, reflect upon the way the world’s five largest tech corporations by capitalization are trying to reshape national and global development discourses.

In the next section, we focus on the corporate penetration, and depoliticization, of the policy space, and the reshaping of epistemic infrastructures.

### 3.1. Corporate influence in AI policymaking

Big Tech derives its influence on national policymakers and international development actors from the design and control of the means of datafication, by establishing what is to be seen and in which way, and leveraging algorithmic prediction to build their epistemic hegemony. Besides active lobbying on national governments ‘to shape or remove the law to fit their controllers’ world view’ (Dignam, 2020, p. 46), Big Tech relies on material and discursive assemblages to influence the public perception of AI and, in the Global South, frame it in developmental terms. So, for instance, setting asides their competition for market shares, Microsoft, IBM, Amazon, Meta/Facebook, and Alphabet/Google participate in the Partnership on AI, an organization established in 2016 to steer the conversation on the societal challenges of AI (and how Big Tech themselves is better positioned than politicians to address them) (Hern, 2016). This assemblage includes organizations that, although not directly related to Big Tech, leverage their access to policymakers, particularly in the Global South, to advance disruptive narratives of digital transformation. In these narratives, the epicenter of this AI-led revolution is Silicon Valley but it radiates outwards. For instance, in 2017, the World Economic Forum (WEF) launched the Center for the Fourth Industrial Revolution (C4IR), in San Francisco.^5^ While the WEF is based in Geneva, the geographic proximity of its C4IR to Silicon Valley signaled to policymakers globally the centrality of US-based tech giants in this announced revolution. As explained by a top manager of the organization, the center's mission was to ‘shape the trajectory of new and emerging technologies, specifically from a governance perspective.’^6^ However, they soon ‘realise[d] that there is a big gap between how fast technologies are developing and how quickly the governance parameters are shaping up.’^7^

After focusing on the broader governance aspects of AI, in 2019 the WEF C4IR started expanding worldwide, consolidating its presence in 15 countries over the next three years to interface the center's mission with local policy agendas, ‘focusing on the application side of AI.’^8^ Two centers were established in Africa: South Africa and Rwanda. These centers have significant autonomy in managing their portfolios of technology policy projects, coordinating with headquarters to ‘leverage the forum's extensive network to get together a community of like-minded people, experts, to develop frameworks and pilots.’^9^ Moreover, at the beginning of 2021, the WEF C4IR launched the Global AI Action Alliance in partnership, among others, with corporations (IBM, Microsoft), international organizations (OECD, UNICEF, UNESCO, International Trade Union Confederation – ITUC), universities (Northwestern University, Imperial College London, University of Toronto) and think tanks (Equal AI, Institute of AI). According to one C4IR executive, the purpose of this alliance is to ‘bring these [frameworks and pilots] to scale. We scan the horizon, we speak to our partners, we look out for interesting opportunities to develop consistently with our criteria, including the neutrality of the project, its multi-stakeholder design and scalability.’^10^ The WEF C4IR shares its role as ‘lynchpin of discussion’ (Anderson, 2017) around AI with consultancies such as McKinsey, BCG (Boston Consulting Group), PwC (PriceWaterHouseCoopers) and Deloitte, which are devoting considerable efforts to shape the policy trajectory of the 4IR agenda (Morgan, 2019; see also Bughin et al., 2018; Hawksworth et al., 2018; Manyika et al., 2016). As an international consultant explained,

Our role is really to help clients understand what the narrative is, understand what AI is […], what adequate policies would look like, and what we need an AI strategy for. I'd say [our job] is really about helping government that can't make those decisions and figure out what it means.^11^

According to this vision, digital firms play a leading role in shaping the regulatory framework that defines what ‘trusted, transparent and inclusive AI systems,’ according to the Global AI Action Alliance, look like, problematizing crucial developmental issues and deploying technology solutions. A representative of another international consultancy laid out the relevance of this approach when they pointed out that

you can write a beautiful national AI strategy on paper. But it's not just about writing that strategy. It's about actually putting it into practice […] So if you're a developing country, including countries in Africa, and if you want to write national a strategy, it's also about the resources at your disposal actually to implement it.^12^

The corporate attempts to capture the policy space in the Global South are problematic for two main reasons. The first is the excessive focus on outcomes at the expense of accountability; the second is an overreliance on a handful of tech giants. According to interviewees working for think tanks and consultancies, in the conversation between governments and tech firms the emphasis is often placed on the results rather than on the processes and their corollary of ethical concerns.^13^ In the words of a policy specialist working for a global consultancy firm,  

If we’re talking about developing country, and I can definitely speak on behalf of India, their priority still is the application side of the technology. If you go to and speak to even the Department of Science and Technology in India, they'll be like, sure, ethics and governance are important. But our priority is to roll out this application. Right? If you're rolling out an application on artificial intelligence for agriculture, they'll be like, we want this to impact the lives of farmers first. And then we can talk about ethics and governance.^14^

Moreover, while some influential think tanks describe loose and business-friendly data regulation as a win-win game for both the public and the private sector, other analysts and representatives of small-scale tech firms are more cautious.^15^ On the one hand, as suggested by policymakers and civil society organizations, there is a wide capability gap between public and private actors on the value of data, with the latter being widely seen as ahead of the game when it comes to incorporating data within national development strategies.^16^ On the other, Big Tech has a competitive edge over small tech firms and, driven by monopolistic tendencies, pursues a strategy of proprietary lock-in through digital platforms.^17^

### 3.2. Shaping epistemic infrastructures

Over at least the last decade, Big Tech has continuously rolled out projects to expand connectivity in Africa. These include Microsoft’s Project Mawingu, connecting schools, health facilities and government buildings in Kenya’s Laikipia County, or Google’s now defunct Loon, based on a light infrastructure of balloons, or more recently Facebook’s 2Africa project (formerly project Simba), which seeks to encircle Africa with a new network of fiber cables. As many of our interviewees indicated, the interest in harnessing digital technologies during humanitarian crises gained momentum in the wake of the 2010 Haiti earthquake^18^ (see also Dugdale et al., 2012). The humanitarian disaster on the Caribbean Island presented an opportunity for US Big Tech to burnish its CSR credentials and experiment with the rapid rollout of ambitious new projects. Google’s charity initiative, Google.org, for instance, dispatched a small team and hardware to Haiti to help bring back connectivity and created a page to offer Haitians real-time updates on the relief efforts.^19^ IBM provided 2 million USD in donations and partnered with humanitarian organizations to map the aid logistics and create a mobile data center.^20^ Following the Haiti earthquake, connectivity started featuring prominently on the humanitarian response checklist. NetHope, a non-profit organization providing IT solutions in critical settings, expanded its partnership with the private sector. Initiatives such as DadaabNet, designed to link and provide connectivity to the humanitarian personnel in Dadaab refugee camp, in Kenya, set a blueprint for most AI4SG corporate interventions in the humanitarian sector in which tech firms provided services to aid agencies rather than to refugees.^21^

These projects were, and continue to be, based on the assumption exemplified by the project manager of a Big Tech corporation in an interview when they argued

if you give the community internet capability, they can do businesses – any possibility you can think of, health, agriculture, selling their produce. There are a million use cases for the internet; the discussion is on how we ensure this technology works.^22^

Focusing not just on the ‘unconnected,’ but also the ‘underconnected,’ the tech firms ‘covering the last mile’ in internet provision have been driven not only by corporate social responsibility (CSR), but also by commercial concerns. As explained by the executive of another Big Tech actor, the corporation’s goal in increasing connectivity, including through a Free Basics initiative, was to ‘provide a more stable platform to access their services.’^23^

The growing corporatization of humanitarianism and development coincides with a shift from connectivity to data analytics and the construction of proprietary epistemic infrastructures. The depoliticization of the policy space is thus entwined with the ‘platformization of infrastructure’ (Plantin et al., 2016) which focuses on Big Tech’s development of infrastructure to extend access to their platforms (or get more users), as digital technologies are ‘making possible lower cost, more dynamic, and more competitive alternatives to governmental or quasi-governmental monopoly infrastructures, in exchange for a transfer of wealth and responsibility to private enterprises’ (Plantin et al., 2016). Recent interdisciplinary research has focused on the ‘platformization of development’ (Heeks, 2009; Madon & Schoemaker, 2021; Mann & Iazzolino, 2021), meaning the strategic rollout of digital platforms to deliver services on behalf of the state. Digital platforms have become a favorite topic of empirical and theoretical reflection across multiple disciplines (Srnicek, 2017) because of the variegate issues they raise, ranging from the opacity of their operations to their capacity to displace government’s prerogatives, or what the legal scholar Frank Pasquale (2018) calls ‘functional sovereignty’ regarding the way tech giants like Amazon or Google are de facto encroaching state regulators in managing markets (Atal, 2020). By embodying a promise of greater efficiency and cost-saving, digital platforms seek to fill an institutional void (Heeks et al., 2021) in several African countries still reeling from the rollback of the state during the 1980s Structural Adjustment Programs or supporting humanitarian agencies amidst funding shortages. This is the case, for instance, of the public-private efforts to datafy city management in smart city initiatives rolled out by Huawei across Africa, the construction of networks of weather stations designed and implemented by Syngenta Foundation in East Africa, or the myriad of digital agricultural platforms providing extension services to smallholder farmers that are being launched by both start-ups and large corporations in West and East Africa (Iazzolino & Mann, 2019), or the data analytics platforms deployed in the humanitarian sector.

Platformization thus serves the purpose of sourcing the data that make visibility possible. Platform operators derive their influence on national policymakers and international development actors from the design and control of the means of datafication, by establishing what is to be seen and in which way. The datafication of public services, including social protection programs (Masiero & Das, 2019), digital identity verification, tax collection, and security, enables private corporations to strategically position themselves as a ‘difficult-to-displace intermediary (or even a critical infrastructure)’ (Milan et al., 2021, p. 388). Public infrastructures, including the provision of water or electricity, are increasingly embedded into broader data capture apparatuses. User engagement with these data apparatuses produce digital footprints, enabling a greater level of granularity and strengthening the monitoring power of the platform operators. Couldry and Mejias (2019) use the notion of ‘social caching’ as ‘a new form of knowledge about the social world based on the capture of personal data and its storage for later profitable use’ (19), drawing parallels with colonial patterns of dispossession and extraction (see also Ricaurte, 2019). The ‘colonial gaze’ is therefore updated and magnified by the ‘algorithmic gaze’ – or the ‘algorithms’ ability to characterize, conceptualize, and affect users’ (Kotliar, 2020). Defined in very broad strokes as a ‘systematic method composed of different steps’ (Jaton, 2021), the algorithm is an analytical/predictive model that learns ‘by inductively generating outputs that are contingent on their input data’, thus ‘engaging experimentally with the world’ (Amoore, 2020, p. 12). Digital platforms make this ‘engagement with the world’ possible by integrating multiple data ingestion points and bound to the algorithm in a feedback loop, in which the constant data extraction enables the fine-tuning of this latter.

In general, increasing emphasis on digital platforms stems from both business actors’ and development practitioners’ awareness of the value of data as a resource to glean a more granular view of the context of implementation, fulfilling donors’ or shareholders’ need for evidence and training predictive models.^24^ The construction of digital platforms embedding multiple data ingestion points is a technopolitical strategy through which corporate actors capture and problematize, according to their priorities and logic, social, economic and political relationships in different contexts. The implications of this corporate-led strategy to penetrate African economies and occupy the space between the state and the citizens are evident in the private sector’s lobbying efforts on governments to digitize government–to-person (G2P) payments, including social protection transfers (Iazzolino, 2018). Digital payment proponents portray the construction of digital payment ecosystems as a win-win for both the state and the private sector (Almazan & Vonthron, 2014), emphasizing the advantages for regulators and state agencies in terms of the use of data trails to police opaque channels, enforce financial integrity and improve tax collection (De Koker & Jentzsch, 2013; Demirguc-Kunt et al., 2015); and for financial service providers in terms of extracting economic value from user-generated data to improve market segmentation for risk assessment (Aitken, 2017).

Influential think tanks like CGAP and Better than Cash Alliance celebrate the money-saving benefits of the externalization of social protection programs, with examples from South Africa, where administrative costs of delivering South African Social Security Agency (SASSA) grants were almost halved when the payments were rerouted through commercial bank accounts, accessible through debit cards; or policy innovations to divert citizens from cash, such as ‘Cashless Nigeria,’ launched by the Central Bank of Nigeria (CBN) in 2012 to establish a daily limit on cash withdrawals and scale up the deployment of point-of-service (POS) terminals (Loeb, 2015). These initiatives emanate from broader corporate-friendly policies poised between surveillance and inclusion. This corporate-led digital re-infrastructuring presents specific risks in humanitarian contexts in which users are unable to opt out of engaging with this data ecosystem because it would be highly costly in terms of well-being or even survival (Iazzolino, 2021). In this case, our concept of depoliticization through datafication may seem aligned with the neutral image that humanitarian organizations are willing to project. And yet, while humanitarian actors stress their insulation from politics, scholars in critical humanitarianism have dismissed these claims as misleading (Pallister-Wilkins, 2020). In fact, AI analytics and datafication further obfuscate the political entanglement of care and surveillance behind a nonpolitical veneer.

### 3.3. Data and predictive power

As data extractive infrastructures, digital platforms play a critical role in fine-tuning algorithms, as highlighted by AI scholars (Birhane, 2019; Nowotny, 2021) who stress the rising relevance of prediction in, among other fields, policing (Degeling & Berendt, 2018; Karppi, 2018), welfare (Eubanks, 2018) and climate change (Machen & Nost, 2021). Big Tech views AI4SG as a safe space to train their predictive models by partnering with governments and international organizations wishing to burnish their innovative credentials and using the data they collect from their population of concern as algorithmic fodder.^25^ These partnerships are particularly relevant in the emerging field of ‘anticipatory humanitarian action,’ an approach in which open and proprietary datasets are leveraged to train predictive models, anticipate the likely trajectories of humanitarian crises and strengthen the preparedness of aid agencies and national governments. Currently, most data-driven anticipatory humanitarian action interventions focus on humanitarian crises induced by climate hazards because of the awareness that early responses to conflicts and atrocities come with greater risks and challenges, particularly when navigating complex political contexts (Iazzolino, McGeer, & Stremlau, 2022). However, this field is quickly evolving in light of the growing interest from policymakers and humanitarian practitioners, technological advances in AI/ML and increased involvement of corporate actors such as Google, Meta, and IBM.

A telling example is the Foresight project, launched by the Danish Refugee Council (DRC), a humanitarian organization partnering with IBM to leverage data analytics to generate insights on displacement trajectories in Myanmar and Afghanistan. One of the project managers explained that one of the reasons given by IBM to develop forecast-based humanitarian interventions was ‘to figure out how their model can be improved’ and how to ‘make their prediction more accurate’^26^. This partnership can thus be viewed through what Amoore (2020) calls the ‘experimental space of play,’ in which a model of the world is iteratively updated through a ‘trial and error process of building models and checking the performance of the models when particular features are included or excluded’ (Kelleher, 2019, p. 24 in Amoore, 2020).

Although ‘AI has lowered the cost of prediction,’^27^ not all the actors, whether public or private, have the human or technical capability of ‘prospecting,’ or rendering the data they collect ‘amenable to processing with the aid of analytics tools’ (Hansen & Borch, 2022). By locking in small digital firms, dominant platform operators – telecoms like Kenya’s Safaricom and other tech companies – extract rents ‘both “direct” (i.e. fees, charges) and “indirect” (i.e. derived from the capture and analysis of user-generated data)’ (Langley & Leyshon, 2022). In fact, notwithstanding the hype on AI, most tech firms and start-ups operating in the African digital space and without in-house AI resources, rely on platforms like Microsoft Cognitive Services, Google AI or IBM Watson to use artificial intelligence as a service (AIaaS) (Gwagwa et al., 2020) and most developers of African AI start-ups focus on developing and using AI for e-commerce and data analytics (Gwagwa et al., 2020, p. 9). The concept of AIaaS refers to ‘off-the-shelf’ AI services, accessible on-demand via subscription. As the executive of a global tech company in Kenya points out,

‘the problem with AI is that you have to know what kind of tools you need in order to develop a solid language model. And so you can charge people for usage. Big companies tell small companies: I'll give you access to the core and you can build your own product. And hopefully you do it on our cloud, so that you can we can charge you an arm and a leg for that.’^28^

As suggested by some interviewees, Big Tech may leverage AI4SG to showcase their capacities and establish trust relationships with governments and state agencies. As vividly explained by the former research director of a major corporation working in East Africa, 

‘I'm a tech giant and I go to country X to help them build a model for flood forecasting using satellite imagery. I don't think any country would say no to that. However, this is where the real question of influence lies. Flood forecasting becomes like an entry point. Because, ultimately, your goal as a private sector company is to have business. And if you can demonstrate efficacy with this one user case, you can then also tell them that hey, now you've seen how good we are with our cloud services and our computing capacity. […] This is the concern that some civil society people have raised, that even when tech companies are saying that some of these models will be open source, does that open them the doorway for them to make the government more dependent on them?’^29^

The constellation of Big Tech, international organizations and consultancies that we have described advances this AI-driven solutionist approach against global inequalities in state capacity to invest in research and development, displacing firmly entrenched corporate giants, the legal dominance of the West. As pointed out by a policy expert of a leading consultancy firm,

‘access to computing resources for training and running these AI models is not something every country can afford. Now, if you're the US, you could spend like 6 billion. Do other countries have those kinds of resources?’^30^

Other interviewees underlined that the uneven access to resources and expertise in AI technologies is not just a divide along the North–South axis. Still, it highlights the hegemonic role of a few large tech giants that ‘have those kinds of resources to build out those models.^31^’

Although the recent pandemic has boosted the interest in anticipating future outbreaks and, in general, generating foresights, critics are already drawing attention to the epistemic and ethical downside of this approach, as well as the limits.^32^ Perdomo et al. (2020), for instance, highlight the risk of performative predictions, in which ‘predictive models can trigger actions that influence the outcome they aim to predict.’ Besides, data scientists are stressing the need to identify built-in biases and mitigate the opacity of the computation processes leading to outcomes that might turn harmful for specific segments of the population. While in the Global North regulators and legal scholars are discussing the ‘status of algorithms in law,’ or how to make algorithms a legal entity (Koshiyama et al., 2021). Most countries in the Global South, and not exclusively, may lack teeth to enforce algorithmic auditing, or ‘the research and practice of assessing, mitigating, and assuring an algorithm’s safety, legality, and ethics’ (Koshiyama et al., 2021).

Moreover, the cost of prediction translates into negative environmental externalities that AI4SG champions are only partially accounting for. The boom of Generative AI, which sees applications such as Chat GPT producing outputs on the basis of inputs that have been processed and used to train large language models (LLM), entails an expanding environmental footprint. Despite the challenges of calculating the energy cost of specific AI models, Patterson et al. (2021) have estimated that the training of GTP-3 required 1,287 MWh and produces over 550 tons of carbon dioxide equivalent. In perspective, this amount equals 550 roundtrips between New York and San Francisco taken by a single person (Stokel-Walker, 2023). As the volume of data created worldwide is expected to reach 181 zettabytes by 2025 (IDC, 2021), the issue of data storage is drawing interest not only from corporate actors and investment funds, national governments concerned about data soveriegnty, but also from international policymakers and environmental activists. While Big Tech, telecom operators and real estate firms are announcing the construction of new hyperscale and co-location data centers across Africa, and South Africa and Kenya in particular (The Economist, 2021), to meet the rising data storage demand and local policymakers’ emphasis on digital sovereignty, grassroots opposition is growing in established data center markets in US (Cappella, 2023) and Europe (Rone, 2022, 2023), and even in emerging markets in Latin America (Lehuedé, 2022) contesting the corporate appropriation of water resources and raising issues of energy justice related to the uneven access to the power grid. These mobilisations from below highlight the politically charged nature of data infrastructures, suggesting that, besides the hype on how AI and the 4IR can support sustainability, a conversation on whether the hegemonic approach to datafication is sustainable is warranted.

## 4. Conclusions

This article has argued that by not only supporting, but by actively advancing, the AI4SG and 4IR discourses to place AI and datafication at the center of the development agenda, tech corporations seek to create new opportunities for rent extraction. To achieve this, they attempt to locate themselves in the space between the state, the citizens, humanitarian organizations, and the recipients of aid as hard-to-displace partners. By black-boxing decision-making processes, the firms behind the design, management and maintenance of digital platforms aspire to control the construction of data as an object of power and knowledge (Ruppert et al., 2017). Despite their highly political and ideological agendas, AI4SG and 4IR attempt to be a form of ‘depoliticization through datafication’ in which politics is removed from scrutiny.

Against the quickly shifting backdrop of digital development, initiatives by policymakers and civil society organizations are trying to wrestle the control of data infrastructures from foreign tech firms or make them more accountable. For instance, in recent years, countries including Nigeria, Rwanda, Ethiopia, and Senegal have put the issue of digital sovereignty on the political agenda, not only passing data localization laws that require firms and international agencies to process and store locally all or some types of data collected inside the country, but setting up publicly funded AI research centers and tightening the control over local data infrastructures, including data centers. At the same time, calls for decolonizing AI are being actively advanced in the field by organizations such as South Africa-based Masakhane (which features Microsoft and Mozilla, financially supported by Alphabet/Google).

Further research is required to explore the variety of ways in which African governments are negotiating with tech firms about the incorporation of AI and big data in their national development strategies, as such arrangements are not always transparent or readily accessible. While much of our discussion in this paper has focused on large US-based tech companies, this also highlights the different approaches of US, European and Chinese corporations to secure partnerships with African governments, and how these latter seek to navigate the difference at their advantage to achieve national security goals and economic growth, or a combination of both. There is also a need to examine how activists and data scientists in the Global South are developing subaltern approaches to datafication and AI.

Finally, the growing attention paid by local advocacy organizations to data rights offers some indication that the demand for greater accountability in data and AI assemblages is becoming a growing concern for more citizens. The extent to which data rights issues remain elite-driven, and the purview of internationally-supported civil society groups in capital cities, or whether citizens begin to demand protections in mass, remains to be seen.

As this article has argued, the corporatization of the AI4D agenda threatens not only how government and policy actors conceptualize what exactly ‘development’ means in the context of 4IR but also deeply skews indicators and values. It changes the discourse and language- and while some of this may be for the better, unless the technological capabilities of governments and policy actors are rapidly upskilled, the outsourcing of AI4D will only accelerate, leaving the public sector and state further behind. Lastly, the influence of Big Tech through AI4SG is a theme that global development scholars need to account for against a background of dwindling public funding for development research and datafication of knowledge infrastructures.

We have been careful to stress that the AI4SG programs currently rolled out emanate from the attempts of a constellation of corporations, international organizations and philanthro-capitalist foundations that do not always share the same goals, or motivations. However, together they are having an outsized impact shaping the policy conversation around AI and development, influencing the regulatory framework to facilitate the expansion of data infrastructures, and asserting a hegemony of technical experts in charge of anticipating the future. Yet, future has not to be anticipated, but made. And this article is a call for acknowledging the political nature of future-making. ICT4D can differentiate itself from AI4SG by continuing to put stress on the D of Development and keeping the spotlight on the political nature of the concept – a nature that the SG of Social Good is concealing behind a technocratic veneer. This is a conversation that ICT4D scholars and practitioners need to have sooner rather than later.

## Notes

1 The term Big Tech is commonly used in the literature, including in this article, with reference to the world’s largest tech firms by capitalisation: Apple, Microsoft, Alphabet (Google), Amazon, and Meta Platforms (Facebook). In this paper, we include in this definition other large technological corporations that are active in the AI4SG space.

2 These interviews were conducted as part of the European Research Council (ERC) under the European Union's Horizon 2020 research and innovation programme-funded ConflictNET project (grant no 716686).

3 Interview with Data scientist, Project manager, Big Tech corporation 2.

4 Interview with Data scientist, Big Tech corporation 2.

5 Interview with Lead, AI & Climate Technology, International consultancy firm 2.

6 Interview with Lead, AI & Climate Technology, International consultancy firm 2.

7 Interview with Lead, AI & Climate Technology, International consultancy 2 firm.

8 Interview with Managing Director, International consultancy firm 2.

9 Interview with Managing Director, International consultancy firm 2.

10 Interview with Lead, AI & Climate Technology, International consultancy firm 2.

11 Interview with Policy specialist, international consultancy firm 1.

12 Interview with Consultant, international consultancy firm 3.

13 Interviews with Project manager, Big Tech corporation 2; Policy specialist, International NGO 3; Director, Civic organisation 1.

14 Interview with Policy specialist, international consultancy firm 1.

15 Interview with Project manager, Think tank 1; CEO, Tech company 1; Lead data analyst, Tech company 1; CTO, Tech company 4; Project manager, Civic organisation 2; Project manager, Development agency.

16 Interview with Policy Lab innovation coordinator, Government agency 1; Executive, Think tank 2.

17 Interview with Project manager, Think tank 1; CEO, Tech company 1; Lead data analyst, Tech company 1; CEO, Tech company 5; AI advisor, Development agency; Innovation and Digital Solution Advisor, Development agency.

18 Interview with Project manager, International NGO 1; Innovation officer, UN agency 3; Innovation officer, UN agency 3.

19 Project manager, Big Tech corporation 1; – Project manager, International NGO 2; Innovation officer, UN agency 3.

20 Interviews with Project manager, Big Tech corporation 2.

21 Interview with Data scientist, UN agency 2; project manager, International NGO 1.

22 Interview with Project manager, Big Tech corporation 1.

23 Interview with Executive Big Tech corporation 3.

24 Interview with Data scientist, UN agency 1; Strategy Lead, Project manager, Policy specialist, international consultancy firm 2.

25 Interview with Global advisor/Senior analyst, International NGO 4; Innovation officer, International NGO 8.

26 Interview with Global advisor/Senior analyst, International NGO 4.

27 Interview with Data scientist, Big tech corporation 2.

28 Interview with Executive, Big Tech corporation 3.

29 Interview with Policy manager, Big Tech corporation 2.

30 Interview with Policy specialist, international consultancy firm 1.

31 Interview with Solution developer, Tech company 2.

32 Interview with Policy specialist, International NGO5; Innovation officer, International NGO 8; Director, Civic organisation 1.
